# HIV/AIDS presenting with stroke-like features caused by cerebral Nocardia abscesses: a case report

**DOI:** 10.1186/s12883-015-0437-7

**Published:** 2015-10-07

**Authors:** James Stefaniak

**Affiliations:** University of Cambridge School of Clinical Medicine, Cambridge, UK

**Keywords:** Human Immunodeficiency Virus, Acquired Immune Deficiency Syndrome, Opportunistic, Nocardia, Abscess, Stroke

## Abstract

**Background:**

Immunosuppression in Human Immunodeficiency Virus can predispose to opportunistic infections of the central nervous system and can be life threatening without early recognition and management. This can be delayed in undiagnosed Human Immunodeficiency Virus. The present article is the only case report in the literature to describe a first presentation of Acquired Immune Deficiency Syndrome as cerebral Nocardia abscesses that were initially treated as a stroke.

**Case presentation:**

A previously well 59 year old Caucasian man presented with sudden onset of left sided hemiparesis and sensory change, right sided headache, diplopia and confusion. The patient was initially treated as a stroke but was eventually found to have pulmonary and cerebral Nocardia abscesses secondary to a new diagnosis of Human Immunodeficiency Virus/Acquired Immune Deficiency Syndrome.

**Conclusion:**

Human Immunodeficiency Virus infection can produce a variety of neurological presentations with the added possibility of multiple pathological processes being present simultaneously. This is only further complicated in instances, such as the present case, when Human Immunodeficiency Virus infection has not yet been diagnosed. It is therefore imperative that appropriate neuroimaging is done at an early stage to ensure timely initiation of appropriate therapy. Cerebral Nocardia abscesses are a serious and potentially life threatening complication of Human Immunodeficiency Virus.

## Background

Immunosuppression secondary to Human Immunodeficiency Virus (HIV) can predispose to opportunistic infections of the central nervous system (CNS). Many such CNS infections are Acquired Immune Deficiency Syndrome (AIDS)-defining illnesses and include toxoplasmosis, cryptococcal meningitis, tuberculosis, cytomegalovirus and Progressive Multifocal Leukoencephalopathy (PML) secondary to JC virus reactivation [1]. Nocardia is a gram positive bacterium that predominantly infects immunocompromised hosts and affects both pulmonary and extra-pulmonary sites, but is relatively rarer than PML, toxoplasmosis and cryptococcal meningitis as a cause of opportunistic CNS infections in HIV/AIDS [2]. In a 1994 case series of thirty patients with nocardiosis in HIV/AIDS only one patient over a four year period had simultaneous pulmonary and extra-pulmonary nocardiosis [3]. Indeed, the present authors could only find one case report of intracranial nocardiosis in the setting of AIDS published in the past decade in which a man with previously diagnosed HIV presented with amnesia, weakness, headaches and fever and was found to have multiple ring enhancing lesions in his frontoparietal cortices bilaterally [4]. Indeed, many CNS opportunistic infections present with similarly non-specific features such as fever, confusion and lethargy. Such non-specific presentations can delay recognition and appropriate treatment, particularly in patients with undiagnosed HIV/AIDS. Furthermore, there are multiple differentials of cerebral ring enhancing lesions in HIV/AIDS including toxoplasmosis, tuberculosis, fungal abscess and primary CNS lymphoma [5]; definitive diagnosis relies on biopsy, and so consideration of Nocardia in the initial differential diagnosis is essential to prevent delays in initiation of treatment for this rare but potentially fatal CNS infection.

The present article reports the case of a previously well 59 year old man presenting with sudden onset of left sided hemiparesis and sensory change, right sided headache, diplopia and confusion who was initially treated as a stroke but was eventually found to have pulmonary and cerebral Nocardia abscesses secondary to a new diagnosis of HIV/AIDS. This is the only case report in the literature to describe a first presentation of HIV as cerebral Nocardia abscesses that were initially treated as a stroke.

## Case presentation

A 59 year old Caucasian retired sailor with past medical history notable only for hypertension, who was not on regular medication and who had a significant alcohol and smoking history was admitted to the emergency department. He described sudden onset left arm and leg weakness 6 days previously with ‘pins and needles’ and a ‘dragging’ sensation in his left leg for 3 days and right sided headache for 5 days that was sharp, constant, kept him awake at night and was worse in the morning and on coughing. He also complained of recent onset diplopia and an episode of fever (self measured temperature 39 °C) 7 days before admission.

On examination he was confused with weakness (Medical Research Council [MRC] power 3/5), drift, mild spasticity and numbness to light touch throughout his left arm and leg. The left knee was hyperreflexic and his left Babinski reflex was positive. His gait was ataxic.

**Fig. 1:**
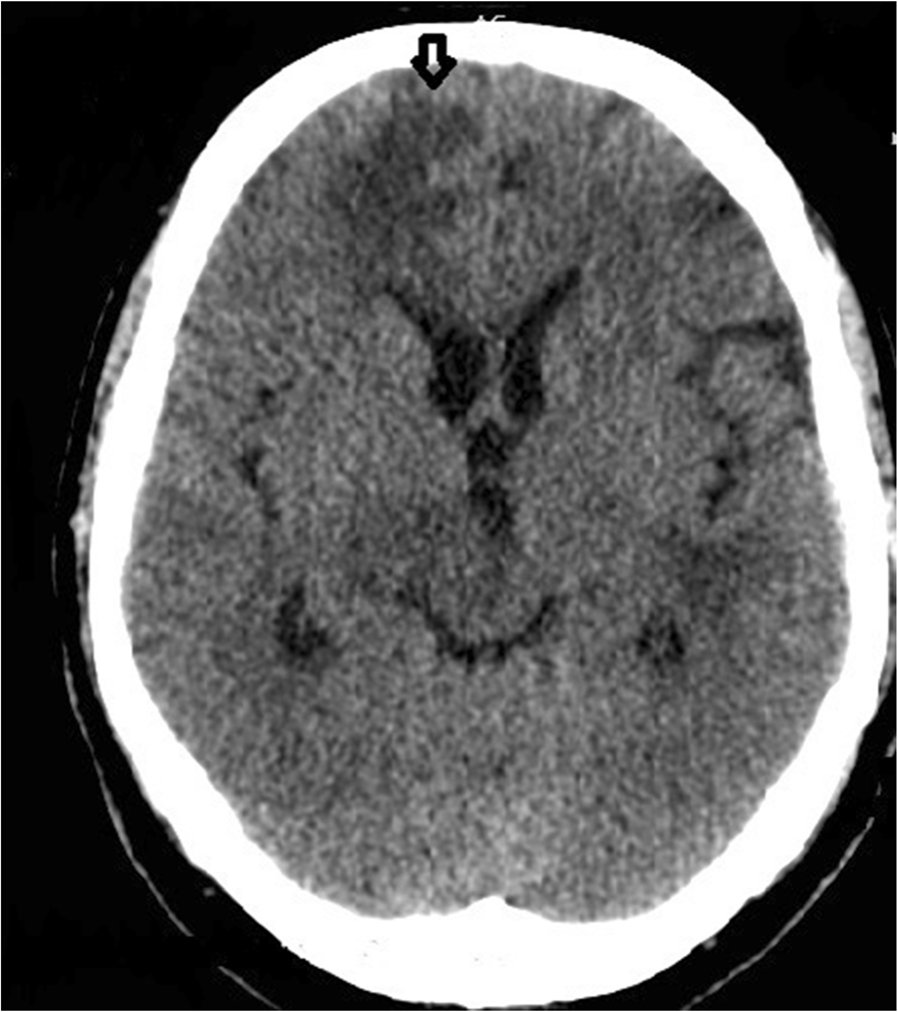
Non-contrast Computed Tomography head. Non-contrast Computed Tomography head taken on admission showing scattered areas of hypodensity involving grey and white matter in the right basal ganglia, bilateral frontal lobes (see arrow) and bilateral parietal lobes

Admission blood results demonstrated a mild macrocytosis (Mean Cell Volume [MCV] 105 fL), neutrophilia (neutrophil count 12 x 10^9^/L), lymphopenia (lymphocyte count 0.39 x 10^9^/L) and hyponatremia (sodium 130 millimolar (mM). An initial non-contrast Computed Tomography (CT) head (see Fig. 1) showed scattered areas of hypodensity involving grey and white matter in the left cerebellar hemisphere, right basal ganglia, bilateral frontal lobes, bilateral parietal lobes and left occipital lobe. Ventricles were undistended and undisplaced, no mass or haemorrhage was seen and the images were reported as being consistent with multiple ischaemic infarcts. The patient was diagnosed as having a stroke with a National Institute of Health Stroke Score (NIHSS) of 3 and started on aspirin, a statin and an angiotensin converting enzyme (ACE) inhibitor.

**Fig. 2:**
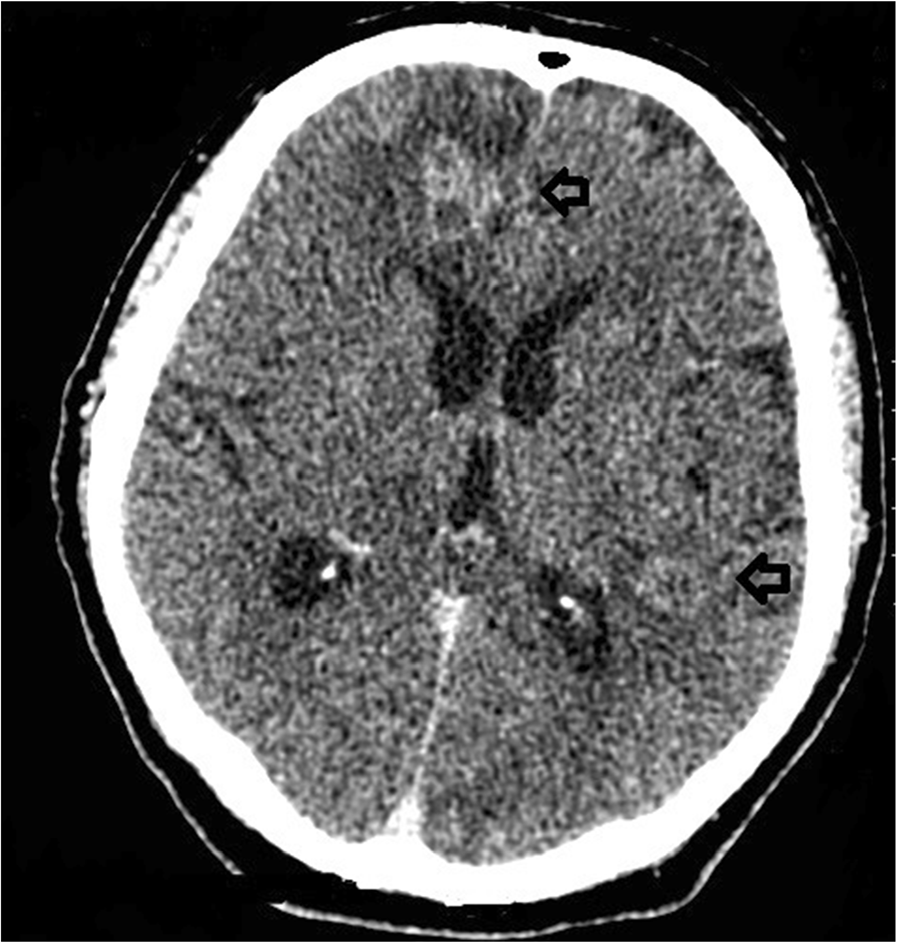
Computed Tomography head with contrast. Computed Tomography head with contrast showing multiple ring enhancing lesions with surrounding oedema (see arrows)

On day 2 of admission the non-contrast CT head report was revised as being suggestive of metastatic lesions with surrounding oedema, raising the possibility of cerebral metastases. It was noted that the hypodensities on the non-contrast CT head demonstrated cortical sparing and were not wedge shaped, suggesting against multiple lacunar infarcts. A CT-chest, abdomen and pelvis and a CT head with contrast were requested for staging of the presumed tumour, and dexamethasone was started for presumed symptomatic cerebral metastases with oedema. Due to patient confusion, this imaging was only obtained whilst anaesthetised on day 6 of admission. The CT head with contrast (see Fig. 2) showed the aforementioned hypodensities to be multiple ring enhancing lesions with surrounding oedema; additional lesions were noted in the brainstem, and the largest lesion was in the right frontal lobe measuring 22 mm in diameter. It was reported that appearances were consistent with disseminated brain metastases but that intracerebral tuberculomas could give a similar appearance. The CT-chest, abdomen and pelvis showed left upper lobe consolidation suggestive of infection, with no evidence of metastases, primary tumour or adenopathy. Blood tests for tumour markers alpha-fetoprotein, cancer antigen 19–9 (CA19-9), carcinoembryonic antigen (CEA) and prostate specific antigen (PSA) were all within normal limits. Prophylactic levetiracetam was started.

**Fig. 3:**
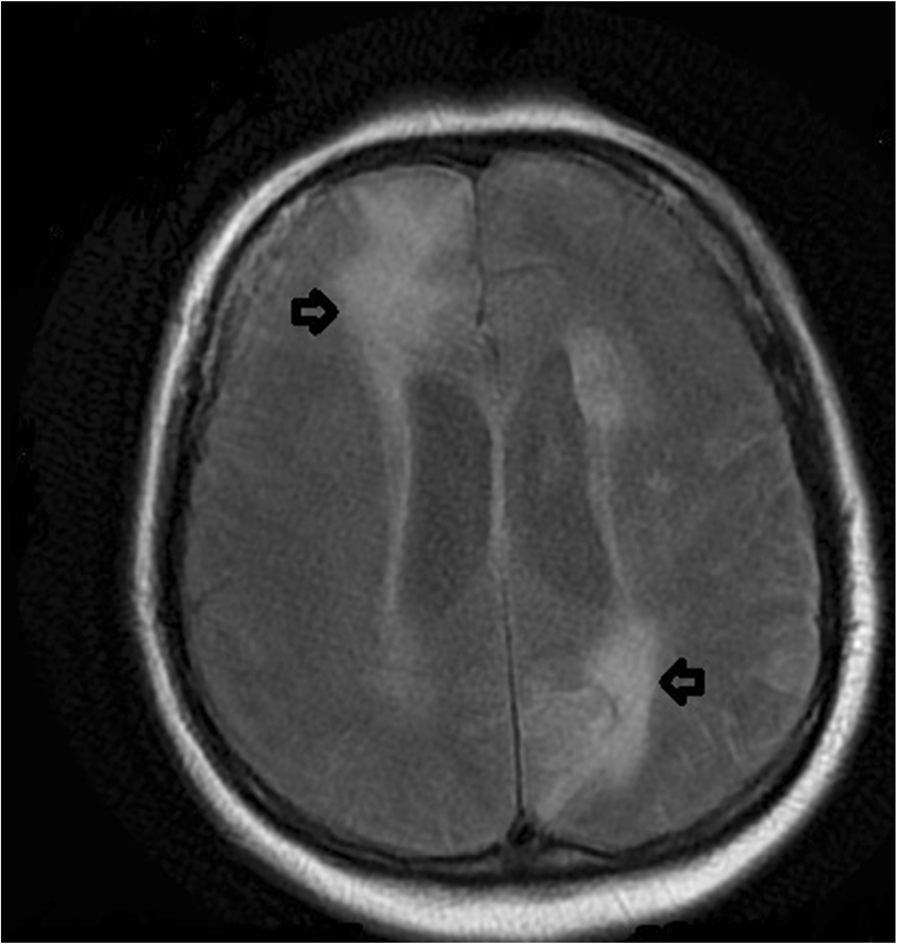
Diffusion Weighted Image-Magnetic Resonance Image. A Diffusion Weighted Magnetic Resonance Image showing multiple lesions with restricted diffusion (see arrows) consistent with multiple abscesses and developing hydrocephalus

A Diffusion Weighted Image-Magnetic Resonance Image (DWI-MRI) with contrast was subsequently obtained (see Fig. 3). This showed multiple lesions with restricted diffusion consistent with multiple abscesses and developing hydrocephalus. A HIV test was requested; serology was positive for HIV-1, HIV-1 ribonucleic acid (RNA) was detected on polymerase chain reaction (PCR) and the patient’s CD4 T cell count was 50/mm^3^. A stereotactic biopsy of the right frontal lobe abscess was performed; PCR of the aspirate demonstrated the presence of Nocardia. PCR on a subsequent Broncho-Alveolar Lavage (BAL) confirmed a diagnosis of pulmonary and cerebral Nocardia. The patient was started on Highly Active Antiretroviral Therapy (HAART) for HIV and cotrimoxazole for pulmonary and cerebral nocardiosis. An External Ventricular Drain (EVD) was inserted due to hydrocephalus; acute bilateral frontal lobe haemorrhages with intraventricular extension on the left were noted on a repeat CT head following neurosurgery. The patient had neurosurgical burr holes and was cared for on a neuro-intensive treatment unit (ITU) for several weeks, but eventually recovered and was discharged. Follow up CT head showed a reduction in size of the previous haematoma, decreased size of abscesses and no hydrocephalus.

## Discussion

HIV infection can produce a variety of neurological presentations at all stages of the illness, with the added possibility of multiple pathological processes being present simultaneously. As well as predisposing to atypical CNS infections, HIV itself can cause HIV-associated dementia, vacuolar myelopathy, distal sensory peripheral neuropathy, mononeuritis multiplex and polymyositis. Combining this with potential side effects of antiretroviral drugs leads to a diagnostic challenge that is only further complicated in instances, such as the present case, when HIV infection has not yet been diagnosed. It is therefore imperative that appropriate imaging is done at an early stage to ensure timely initiation of appropriate therapy. Non-contrast CT heads often have poor specificity in diagnosing intracranial abscesses; even ring-enhancing lesions on contrast-enhanced CT head images have a wide differential diagnosis, including toxoplasmosis, tuberculosis, bacterial and fungal abscess, primary CNS lymphoma and solid tumour metastases with surrounding oedema [5]. It was only after diffusion weighted MRI that intracranial abscesses were thought to be the most likely pathology in the present patient and thus a cause for probable immunosuppression was sought; this was because diffusion restriction favours a diagnosis of intracranial abscess [4]. Definitive diagnosis inevitably requires biopsy of the lesion in question. Whilst Nocardia has historically been diagnosed using conventional culturing techniques [6], validated PCR tests have been developed to rapidly diagnose Nocardia species in tissue samples [6], thus enabling more rapid initiation of definitive therapy. However, it should be borne in mind that up to 15 % of CNS opportunistic infections in HIV/AIDS involve multiple concurrent processes [1]; a failure to respond adequately to definitive therapy should therefore merit further investigations for the possibility of comorbid CNS infections.

## Conclusions

This report describes a case of pulmonary and cerebral Nocardia abscesses secondary to undiagnosed HIV/AIDS that presented with clinical signs of a stroke. This highlights the variety of possible neurological presentations of HIV/AIDS and the appropriate investigations and management of cerebral abscesses.

## Consent

Written informed consent was obtained from the patient for publication of this Case Report and any accompanying images. A copy of the written consent is available for review by the Editor-in-Chief of this journal.

### Ethics

Ethical approval was not required for this Case Report.

## Abbreviations

HIV: Human Immunodeficiency Virus
CNS: Central Nervous System
AIDS: Acquired Immune Deficiency Syndrome
PML: Progressive Multifocal Leukoencephalopathy
MRC: Medical Research Council
MCV: Mean Cell Volume
mM: Millimolar
CT: Computed Tomography
NIHSS: National Institute of Health Stroke Score
ACE: Angiotensin converting enzyme
CEA: Cancer antigen 19–9, CA19-9 carcinoembryonic antigen
PSA: Prostate specific antigen
DWI: Diffusion Weighted Image
MRI: Magnetic Resonance Image
RNA: Ribonucleic acid
PCR: Polymerase chain reaction
BAL: Broncho-Alveolar Lavage
HAART: Highly Active Antiretroviral Therapy
EVD: External Ventricular Drain
ITU: Intensive treatment unit
